# Influence of Smoking Status on Risk of Incident Heart Failure: A Systematic Review and Meta-Analysis of Prospective Cohort Studies

**DOI:** 10.3390/ijerph16152697

**Published:** 2019-07-29

**Authors:** Hyeonju Lee, Youn-Jung Son

**Affiliations:** 1 Department of Nursing, Tongmyong University, Busan 48520, Korea; 2 Red Cross College of Nursing, Chung-Ang University, Seoul 06974, Korea

**Keywords:** smoking, heart failure, incidence, systematic review, meta-analysis

## Abstract

Smoking is a well-known risk factor for atherosclerotic cardiovascular disease. However, there are insufficient data regarding the predictive influence of smoking status on the risk of incident heart failure (HF). This study involved a systematic review and meta-analysis of prospective cohort studies to identify the association of smoking status with incident risk of HF. Peer-reviewed articles published in PubMed, Embase, Web of Science, Cochrane, and CINAHL up to May 2019 were identified. Seven studies, based on 42,759 participants and 4826 HF cases, were included. Pooled hazard ratios (HRs) and their 95% confidence intervals (CI) were estimated using the fixed effects model. Subgroup analyses were conducted to define possible sources of heterogeneity. Current smokers aged 18 years and over had a greater risk of HF incidence compared with non-smokers (never or former smokers) (HR = 1.609, 95% CI, 1.470–1.761). Additionally, former smokers had a greater risk of HF incidence compared with never smokers (HR = 1.209, 95% CI, 1.084–1.348). The present study highlighted that never smokers have more obvious cardiovascular benefits than current or former smokers. Therefore, health professionals should support cessation at the earliest among current smokers and encourage young people and non-smokers not to start smoking.

## 1. Introduction 

The rapid worldwide growth of the aging population has led to an increasing incidence and prevalence of heart failure (HF) [1]. HF is a chronic and progressive illness that is associated with high morbidity and mortality and medical costs owing to frequent hospital readmissions [2]. Moreover, despite advancements in medical and device therapy, HF continues to be associated with adverse outcomes and poor prognosis. The prevention of HF in community-based populations, therefore, is an important public health issue at the regional, national, and global levels [3]. To facilitate effective prevention, early identification of the relevant risk factors is crucial. Accordingly, there is a need to understand the factors influencing the risk of HF incidence.

To date, numerous studies have reported associations between HF and modifiable cardiovascular risk factors including smoking, control of blood pressure and glucose, obesity, physical inactivity due to sedentary lifestyle, and unhealthy diet [3,4,5,6]. Among these, smoking has been known as the variable most strongly associated with HF risk [7,8] through cardiac dysfunction such as vessel constriction, impaired endothelial function, and increased blood pressure [8,9]. 

Observational studies have indicated current smokers to be associated with an increased risk of cardiovascular events and all-cause mortality compared with those who have never smoked [10,11]. According to Ahmed et al.’s study [12], former smokers had a similar risk of incident HF and mortality, as that of never smokers when compared to current smokers. Additionally, Kamimura et al. [8] found that intensity and burden among current and former smokers were associated with higher rates of hospitalization due to incident HF compared with never smokers. On the contrary, one study reported that there was no significant association between smoking status and patients’ health status at the time of enrollment [13]. These results are conflicting and inconsistent. Moreover, little is known about the effect of smoking on the risk of HF incidence in the general population. Recently Aune et al. [14] conducted a meta-analysis of prospective studies regarding the association between smoking and HF risk. However, the review included different types of studies such as retrospective and nested case-control studies, which resulted in clinical heterogeneity. In addition, the authors did not take into account the methodological quality of the included studies. 

Therefore, we performed a systematic review and meta-analysis of solely prospective cohort studies to evaluate the causal relationships between smoking status and incident HF in the general population, adjusting for traditional cardiovascular risk factors such as age, sex, body mass index, hypertension, and diabetes. 

## 2. Materials and Methods 

To perform this systematic review and meta-analysis, we adopted the recommendation of the Meta-analysis of Observational Studies in Epidemiology guidelines [15].

### 2.1. Search Strategies

Two investigators (Y.J. and H.J.) independently performed a search in five databases—PubMed, Embase, Cochrane, Web of Science, and CINAHL—from their inception to May 2019 for prospective cohort studies describing the link between smoking status and HF risk. We used the search terms, “smoking,” “tobacco,” “nicotine,” “cigarette smoking” and “heart failure,” “cardiac failure,” “heart decompensation,” “congestive heart failure,” “chronic heart failure” (MeSH) combined with “cohort studies,” “follow-up studies,” “prospective studies,” “longitudinal studies.” The reference lists of potential articles were thoroughly reviewed. Only articles published in English were considered. Ethical approval was not required for this review because of the use of already published data.

### 2.2. Study Selection

For this review, EndNote (version X7, Thomson Reuters, New York, NY, USA) was utilized to eliminate duplicate references, after which two reviewers conducted a manual cross-check of the titles and abstracts of the remaining articles. The following were the inclusion criteria: (1) population-based prospective cohort studies involving human subjects; (2) assessment of smoking status and HF as the exposure and outcome of interest, respectively; (3) assessment of traditional cardiovascular risk factors (hypertension, diabetes, hypercholesterolemia, obesity, and physical inactivity); (4) participants free of HF at the beginning of the studies; and (6) reporting a summary estimate (hazard ratio (HR) or relative risk (RR)) with confidence intervals (CI). The following study types were excluded: (1) cross-sectional, retrospective, case-control studies, clinical trials, reviews, commentaries, editorials, and letters to the editor and (2) review articles and meta-analyses. 

**Figure 1:**
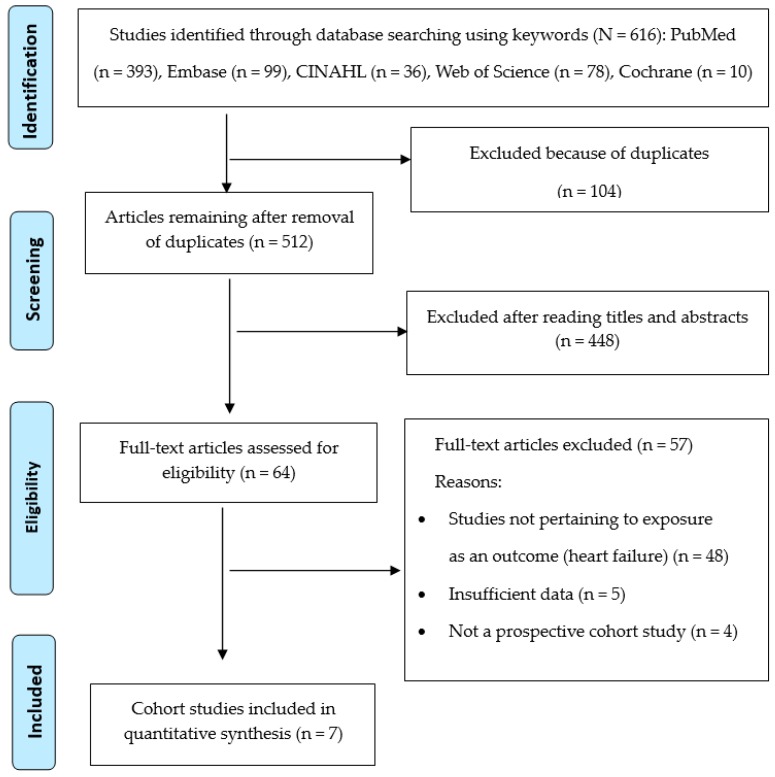
Flowchart of systematic review of literature selection process for the present study.

The criteria for identification of incident HF as the outcome of interest in this review were one or more of the following: (1) medical diagnosis from physician records; (2) evidence of medical therapy for HF including pharmacologic treatment such as diuretics, digitalis, and vasodilators; and (3) a diagnosis with an International Classification of Diseases-Ninth Revision discharge code. The entire description of our screening process is presented in the PRISMA flowchart (Figure 1).

### 2.3. Data Extraction

The data extracted from each study were cross-checked by two independent reviewers. We extracted the following information from each retrieved study: name of the first author, publication year, study location, participants’ ages at baseline, number of cases, size of cohort, duration of follow-up, assessment of smoking status and HF, and covariates that were adjusted for in the multivariable analysis. Any disagreement was resolved by consensus among all reviewers. 

### 2.4. Quality Assessment

We used the Newcastle-Ottawa Scale (NOS) [16] to determine the quality of included prospective cohort studies. The NOS, a representative tool for the meta-analysis of observational studies [16], has been widely used owing to the recommendation of the Cochrane Collaboration [17].

The NOS assesses three quality parameters (selection, comparability, and outcome) divided across eight specific items. There is a series of response options for each item. A star system is used to allow a semi-quantitative assessment of study quality, such that the highest-quality studies are awarded a maximum of one star for each item with the exception of the item related to comparability, which allows the assignment of two stars. Scores range from 0 to 9 and studies with scores of 7 to 9 are considered high quality. Any disagreements in quality assessment were resolved by discussion until a consensus was reached. Ratings regarding the validity of included studies are reported in Table 1. 

### 2.5. Statistical Methodology

We utilized HRs as the risk of HF incidence across studies, and considered RRs as equivalents. We used the fixed effects model owing to low heterogeneity among included studies. We identified Hedges’ Q statistic (statistical significance was set at *p* < 0.05) and the I^2^ statistic with its 95% CI to describe heterogeneity. I^2^ values of 25–50% were considered to indicate low heterogeneity, 50–75% moderate heterogeneity, and > 75% high heterogeneity [23]. 

We conducted subgroup analyses to investigate the sources of the heterogeneity expected in the meta-analysis of cohort studies. Publication bias was assessed using Begg’s rank correlation tests [24] and Egger’s linear regression tests [25]. If publication bias was identified, we adjusted the overall HR with the “trim and fill” method. We assumed that the overall HR was within 10% it was judged that there was no publication bias [26]. 

A sensitivity analysis was also conducted to assess the influence of a single study on the overall HR by omitting one study each time. All statistical analyses were performed using the Comprehensive Meta-Analysis software (version 3.0; Biostat, Englewood, NJ, USA), and all *p* values were two-sided with a significance level of 0.05.

## 3. Results

### 3.1. Literature Search

A total of 616 citations were initially identified from PubMed, Embase, CINAHL, Web of Science, and Cochrane up to May 2019. After the removal of duplicates, 512 citations remained for further assessment. Of these articles, 448 were removed after reviewing the title and abstract, leaving 64 articles for full-text review. Finally, seven articles [9,12,18,19,20,21,22] were included in the meta-analysis.

### 3.2. Description of Included Studies

The characteristics of the included articles are depicted in Table 1. The cohort size of studies ranged from 2125 to 13,643, with a total of 42,759, and the number of HF cases ranged from 100 to 1382, with a total of 4826. The ages of the study participants ranged from 25 to 84 years, and the range of the follow-up period was 9.4 to 20.1 years. By smoking status, participants were classified as current and non-smokers (which included former or never smokers) using self-reports, interviews, and medical records. The quality score evaluated by the NOS was between 7 and 9.

### 3.3. Smoking Status and Incident Risk of HF

**Figure 2:**
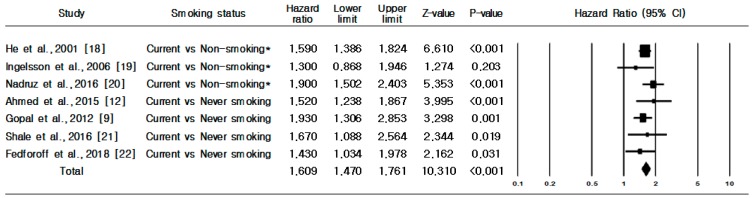
Heart failure incidence in current smokers versus non-smokers or never smokers. Note: * Former and never smoking.

Figure 2 presents the association between smoking status and incident risk of HF. A total of seven prospective cohort studies were included in the analysis to identify the association between risk of HF incidence and current smokers compared with non-smokers (never or former smokers). Compared with non-smokers, current smokers had a greater risk of HF incidence (HR 1.609, 95% CI, 1.470–1.761) (Figure 2). No evidence of heterogeneity was observed (Q = 4.688, *p* = 0.584; I^2^ = 0.0%). Through Begg’s (*p* = 0.764) and Egger’s tests (*p* = 0.992), we confirmed that there was no publication bias regarding current smoking being associated with increased risk of HF incidence.

**Figure 3:**
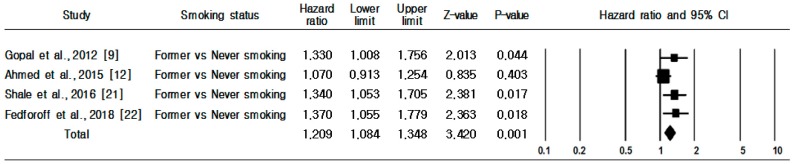
Heart failure incidence in former smokers versus never smokers.

As shown in Figure 3, four prospective cohort studies were used to identify the association between risk of HF incidence and former smokers compared with never smokers. Compared with never smokers, former smokers had a greater HF incidence risk (HR 1.209, 95% CI, 1.084–1.348). No evidence of heterogeneity was observed (Q = 4.309, *p* = 0.230; I^2^ = 30.4%). As Begg’s (*p* = 0.734) and Egger’s (*p* = 0.028) tests identified publication bias, we used the trim and fill method to adjust for it. Accordingly, two studies were added, reducing 7.2% compared to before calibration, and the HR of former smoking and risk of HF incidence was revised to 1.123 (95% CI, 1.029–1.234). 

### 3.4. Subgroup and Sensitivity Analyses

Subgroup analyses by publication year, study location, cohort size, number of cases, follow-up period, and quality assessment score were conducted. Studies with cohort sizes of more than 5000, which included more than 500 HF cases, had conducted follow-ups longer than 15 years, had been published since 2011, and had been conducted in the USA, identified higher HF incidence risk.

In sensitivity analyses, we recalculated the pooled HRs by excluding one study at a time. The pooled HRs ranged from 1.563 (95% CI, 1.417–1.724) to 1.627 (95% CI, 1.483–1.785). The trend was generally similar to the overall analysis. 

## 4. Discussion 

This meta-analysis of seven prospective cohort studies demonstrated that compared with never smokers, current and former smokers were at a greater risk of HF incidence. Most importantly, current smokers had a higher rate of HF incidence than former and never smokers. Our finding was in line with previous studies [8,27] reporting that smoking has negative effects on cardiac function. Namely, tobacco smoking can cause endothelial dysfunction by reducing nitrogen monoxide production, pro-thrombotic conditions, and activating inflammatory routes [28,29,30,31]. These factors, along with the increased amounts of coronary atherosclerosis, may be responsible for the increase in the risk of hypertension [32], coronary heart disease [31] and atrial fibrillation [33,34], potentially contributing to the association of current smokers with higher HF incidence risk noted in the present study [30]. We also observed that past smoking increased the risk of HF incidence compared with never having smoked. A similar study by Anue et al. [14] showed that past smoking (RRs: 1.16) was associated with a lower risk of incident HF compared to current smoking (RRs: 1.75). 

Earlier or long-term smoking cessation may benefit all smokers, regardless of age or amount smoked. Some studies reported that quitting smoking can reduce the risk of death from cardiovascular disease as well as new incidence of cardiovascular disease [35,36,37], and that stopping smoking before age 37 is similar to never having smoked [38]. Accordingly, health professionals should help current smokers attempt smoking cessation as soon as possible. Furthermore, the protection of non-smokers and never smokers through smoke-free environments should be a priority. 

This review showed that there is a lack of empirical evidence on the association between smoking intensity, smoking duration, and HF incidence in population-based cohort studies. In particular, accurate investigations of former smokers, with smoking history accounting for factors such as smoking duration (years), last smoking day, past smoking amount in cigarettes per day, and cumulative exposure (pack-years), are needed to determine whether the risk of HF incidence in this population is relatively lower than among current smokers. Importantly, in most studies included in our review, smokers were not accurately categorized as current, former, and never smokers. In particular, the classification of non-smokers was unclear. Also, the majority of the studies did not investigate former smokers. Moreover, smoking status was mostly measured by self-report or interviews, which can allow smokers to misrepresent their cigarette consumption or even deny it altogether [39]. Thus, biomedical tests are useful in determining the exact relationship between smoking and the risk of a heart attack, and we suggest their usage in further studies.

All seven studies included in the present study were conducted in developed countries and initiated between 1970 and 1997. Studies conducted in developed countries have predicted that quitting smoking is one of the factors that can prevent disease and death around the world [12]. However, there has neither been much research nor adequate smoking cessation services in developing countries, which are expected to have large smoking populations. Studies on the risk of HF incidence are needed for health promotion and disease prevention of the populations in developing countries.

The function of heart increases the size of the left atrium with the progression of aging and promotes left ventricular hypertrophy, which increases the risk of HF incidence [40]. As the participants of the studies included in our review were aged between 25 and 84 years, although we adjusted for age, there is the possibility that the risk of HF incidence could have been influenced by the mean age.

This meta-analysis has several strengths. We analyzed all prior large prospective cohort studies with sufficiently long-term follow-up data for identifying the causal relationship between smoking status and HF incidence risk. All included studies had a quality score of 7 or higher. We considered the studies that included cardiovascular risk factors as confounding factors (hypertension, diabetes, hypercholesterolemia, obesity, and physical inactivity). Lastly, our findings revealed that there was no evidence of publication bias and heterogeneity across included studies. 

However, some limitations of the current study must also be acknowledged. This study represents data derived from observational studies, not clinical trials. Therefore, it cannot directly control for residual or unmeasured confounders although the included studies adjusted for several potential confounders. Only articles written in English were considered in the analysis. If the search had been extended to include studies published in other languages, it is possible that additional relevant trials may have been identified. Next, we did not provide information regarding the risk of HF incidence through comparisons between current and past smoking because we did not have information regarding the age at which former smokers quit smoking, the duration and amount of smoking, and so on. Finally, the results of our review are based on studies from Europe or the United States. Therefore, caution must be exercised in attempting to generalize the results to non-Western populations.

## 5. Conclusions

Our meta-analysis of prospective cohort studies found that current smoking significantly increases the risk of HF incidence among the general adult population. This review highlights that early or long-term smoking cessation may be helpful in preventing the incident HF risk in current smokers. Health professionals should design effective smoking cessation programs based on the tobacco use history of people who want to quit smoking or maintain abstinence from smoking. Policymakers should try to achieve a smoke-free environment so as to shield never smokers from harm. Our findings require confirmation by large-scale prospective cohort studies on the contribution of smoking to HF considering the importance of assessing comprehensive individual smoking or quitting history. 

## Figures and Tables

**Table 1:** Characteristics of studies included.

Authors, (Publication Year)/Location	Age (yrs) at Baseline	Cases/Cohort Size	Follow-up Period (yrs)	Exposure Assessment	Smoking Status Identified	Outcome Assessment	Adjustments for Covariates	Quality Assessment Score
He et al. (2001)/USA [18]	25–74	1382/13,643	19 (average)	Interview	Non-smokers/current smokers	Medical records	Age, race, education, alcohol consumption, physical activity, HTN, SBP, DM, BMI, serum cholesterol, VHD, CAD	9
Ingelsson et al. (2006)/Sweden [19]	50	100/2314	20.1 (median)	Interview	Non-smokers/current smokers	Medical records	HTN, DM, BMI, LVH, MI	8
Gopal et al. (2012)/USA [9]	70–79	231/2125	9.4 (median)	Self-reported	Never/former/current smokers	Medical records	Age, SBP, HR, CAD, LVH, albumin, fasting glucose, creatinine	8
Ahmed et al. (2015)/USA [12]	≥65	931/4482	13	Self-reported	Never/former/current smokers	Medical records	Age, sex, race, education, income, alcohol consumption, ADL, HTN, DM, BMI, CAD, LVH, stroke, AF, PAD, COPD, cancer, left ventricular systolic dysfunction, CRP, ACEIs, serum creatinine, beta-blockers, diuretics	8
Nadruz et al. (2016)/USA [20]	45–64	1496/9649	15	Interview	Never/passive/former/current smokers	Medical records	Age, sex, race, alcohol consumption, HTN, SBP, HR, DM, BMI, SBP, HR, COPD, estimated GFR	9
Sahle et al. (2016)/Australia [21]	84 ± 5	373/6083	10.8 (median)	Medical records	Never/former/current smokers	Medical records	BP, BMI, CVD, estimated GFR	7
Feoforoff et al. (2018)/Finland [22]	27.4–51.3	313/4463	13.8 (median)	Self-reported	Never/former/current smokers	Medical records	Age, sex, HTN, DM, BMI, HDL-cholesterol, TG, HbA_1c_	9

*Note*. HTN = hypertension; SBP = systolic blood pressure; DM = diabetes mellitus; BMI = body mass index; VHD = valvular heart disease; CAD = coronary artery disease; LVH = left ventricular hypertrophy; MI = myocardial infarction; HR = heart rate; ADL = activities of daily living; AF = atrial fibrillation; PAD = peripheral artery disease; COPD = chronic obstructive pulmonary disease; CRP = C-reactive protein; ACEIs = angiotensin-converting enzyme inhibitors; GFR = glomerular filtration rate; HDL = high-density lipoprotein; TG = triacylglycero.

## References

[1] Conrad N., Judge A., Tran J., Mohseni H., Hedgecott D., Crespillo A.P., Allison M., Hemingway H., Cleland J.G., McMurray J.J.V. (2018). Temporal trends and patterns in heart failure incidence: A population-based study of 4 million individuals. Lancet.

[2] Savarese G., Lund L.H. (2017). Global public health burden of heart failure. Card. Fail. Rev..

[3] Avery C.L., Loehr L.R., Baggett C., Chang P.P., Kucharska-Newton A.M., Matsushita K., Rosamond W.D., Heiss G. (2012). The population burden of heart failure attributable to modifiable risk factors. J. Am. Coll. Cardiol..

[4] Dunlay S.M., Weston S.A., Jacobsen S.J., Roger V.L. (2009). Risk factors for heart failure: A population-based case-control study. Am. J. Med..

[5] Khatibzadeh S., Farzadfar F., Oliver J., Ezzati M., Moran A. (2013). Worldwide risk factors for heart failure: A systematic review and pooled analysis. Int. J. Cardiol..

[6] Yang H., Negishi K., Otahal P., Marwick T.H. (2015). Clinical prediction of incident heart failure risk: A systematic review and meta-analysis. Open Heart.

[7] Fleg J.L. (2016). Healthy lifestyle and risk of heart failure: An ounce of prevention well worth the effort. Circ. Heart Fail..

[8] Kamimura D., Cain L.R., Mentz R.J., White W.B., Blaha M.J., DeFilippis A.P., Fox E.R., Rodriguez C.J., Keith R.J., Benjamin E.J. (2018). Cigarette smoking and incident heart failure: Insights from the Jackson Heart Study. Circulation.

[9] Gopal D.M., Kalogeropoulos A.P., Georgiopoulou V., Smith A.L., Bauer D.C., Newman A.B., Kim L., Bibbins-Domingo K., Tindle H., Harris T.B. (2012). Cigarette smoking exposure and heart failure risk in older adults: The health, aging, and body composition study. Am. Heart J..

[10] Mons U., Muezzinler A., Gellert C., Schottker B., Abnet C.C., Bobak M., De Groot L., Freedman N.D., Jansen E., Kee F. (2015). Impact of smoking and smoking cessation on cardiovascular events and mortality among older adults: Meta-analysis of individual participant data from prospective cohort studies of the CHANCES consortium. BMJ.

[11] Thun M.J., Carter B.D., Feskanich D., Freedman N.D., Prentice R., Lopez A.D., Hartge P., Gapstur S.M. (2013). 50-year trends in smoking-related mortality in the United States. N. Engl. Med..

[12] Ahmed A.A., Patel K., Nyaku M.A., Kheirbek R.E., Bittner V., Fonarow G.C., Filippatos G.S., Morgan C.J., Aban I.B., Mujib M. (2015). Risk of heart failure and death after prolonged smoking cessation: Role of amount and duration of prior smoking. Circ. Heart Fail..

[13] Conard M.W., Haddock K., Poston W.S.C., Spertus J.A. (2009). The impact of smoking status on the health status of heart failure patients. Congest. Heart Fail..

[14] Aune D., Schlesinger S., Norat T., Riboli E. (2019). Tobacco smoking and the risk of heart failure: A systematic review and meta-analysis of prospective studies. Eur. J. Prev. Cardiol..

[15] Stroup D.F., Berlin J.A., Morton S.C., Olkin I., Williamson G.D., Rennie D., Moher D., Becker B.J., Sipe T.A., Thacker S.B. (2000). Meta-analysis of observational studies in epidemiology: A proposal for reporting, Meta-analysis of Observational Studies in Epidemiology (MOOSE) group. JAMA.

[16] Wells G.A., Shea B., O’Connell D., Peterson J., Welch V., Losos M., Tugwell P. The Newcastle-Ottawa Scale (NOS) for Assessing the Quality of Nonrandomised Studies in Meta-Analyses. http://www.ohri.ca/programs/clinical_epidemiology/oxford.asp.

[17] Higgins J.P., Green S. (2008). Cochrane Collaboration. Cochrane Handbook for Systematic Reviews of Interventions.

[18] He J., Ogden L.G., Bazzano L.A., Vupputuri S., Loria C., Whelton P.K. (2001). Risk factors for congestive heart failure in US men and women. Arch. Intern. Med..

[19] Ingelsson E., Arnlov J., Lind L., Sundstrom J. (2006). Metabolic syndrome and risk fro heart failure in middle aged mend. Heart.

[20] Nadruz W., Goncalves A., Claggett B., Roca G.Q., Shah A.M., Cheng S., Heiss G., Ballantyne C.M., Solomon S.D. (2016). Influence of cigarette smoking on cardiac biomarkers: The Atherosclerosis Risk in Communities (ARIC) Study. Eur. J. Heart Fail..

[21] Sahle B.W., Owen A.J., Krum H., Reid C.M. (2016). Incidence of heart failure in 6083 elderly hypertensive patients: The Second Australian National Blood Pressure Study (ANBP2). Eur. J. Heart Fail..

[22] Feodoroff M., Harjutsalo V., Forsblom C., Groop P.H. (2018). Dose-dependent effect of smoking on risk of coronary heart disease, heart failure and stroke in individuals with type 1 diabetes. Dibetologia.

[23] Higgins J.P., Thompson S.G. (2002). Quantifying heterogeneity in a meta-analysis. Stat Med..

[24] Begg C.B., Mazumdar M. (1994). Operating characteristics of a rank correlation test for publication bias. Biometrics.

[25] Egger M., Davey Smith G., Schneider M., Minder C. (1997). Bias in meta-analysis detected by a simple, graphical test. BMJ.

[26] Sutton A.J., Duval S.J., Tweedie R.L., Abrams K.R., Jones D.R. (2000). Empirical assessment of effect of publication bias on meta-analyses. BMJ.

[27] Messner B., Bernhard D. (2014). Smoking and cardiovascular disease: Mechanisms of endothelial dysfunction and early atherogenesis. Arter. Thromb. Vasc. Biol..

[28] Barua R.S., Ambrose J.A., Eales-Reynolds L.J., DeVoe M.C., Zervas J.G., Saha D.C. (2001). Dysfunctional endothelial nitric oxide biosynthesis in healthy smokers with impaired endothelium-dependent vasodilatation. Circulation.

[29] Cross C.E., Halliwell B., Borish E.T., Pryor W.A., Ames B.N., Saul R.L., McCord J.M., Harman D. (1987). Oxygen radicals and human disease. Ann. Intern. Med..

[30] FitzGerald G.A., Oates J.A., Nowak J. (1988). Cigarette smoking and hemostatic function. Am. Heart J..

[31] Mendall M.A., Patel P., Asante M., Ballam L., Morris J., Strachan D.P., Camm A.J., Northfield T.C. (1997). Relation of serum cytokine concentrations to cardiovascular risk factors and coronary heart disease. Heart.

[32] Dochi M., Sakata K., Oishi M., Tanaka K., Kobayashi E., Suwazono Y. (2009). Smoking as an independent risk factor for hypertension: A 14-year longitudinal study in male Japanese workers. Tohoku J. Exp. Med..

[33] Zhu W., Yuan P., Shen Y., Wan R., Hong K. (2016). Association of smoking with the risk of incident atrial fibrillation: A meta-analysis of prospective studies. Int. J. Cardiol..

[34] Linneberg A., Jacobsen R.K., Skaaby T., Taylor A.E., Fluharty M.E., Jeppesen J.L., Bjorngaard J.H., Åsvold B.O., Gabrielsen M.E., Campbell A. (2015). Effect of smoking on blood pressure and resting heart rate: A Mendelian randomization meta-analysis in the CARTA Consortium. Circ. Cardiovasc. Genet..

[35] Aune D., Sen A., O’Hartaigh B., Janszky I., Romundstad P.R., Tonstad S., Vatten L.J. (2017). Resting heart rate and the risk of cardiovascular disease, total cancer, and all-cause mortality—A systematic review and dose- response meta-analysis of prospective studies. Nutr. Metab. Cardiovasc. Dis..

[36] Critchley J.A., Capewell S. (2003). Mortality risk reduction associated with smoking cessation in patients with coronary heart disease: A systematic review. JAMA.

[37] Mannan H.R., Stevenson C.E., Peeters A., Walls H.L., McNeil J.J. (2011). Age at quitting smoking as a predictor of risk of cardiovascular disease incidence independent of smoking status, time since quitting and pack-years. BMC Res. Notes..

[38] Jordan H., Hidajat M., Payne N., Adams J., White M., Ben-Shlomo Y. (2017). What are older smokers’ attitudes to quitting and how are they managed in primary care? An analysis of the cross- sectional English Smoking Toolkit Study. BMJ Open.

[39] Shahoumian T.A., Phillips B.R., Backus L.I. (2016). Cigarette smoking, reduction and quit attempts: Prevalence among Veterans with coronary heart disease. Prev. Chronic Dis..

[40] Ferrari A.U., Radaelli A., Centola M. (2003). Invited review: Aging and the cardiovascular system. J. Appl. Physiol..

